# Multi-Proxy Constraint Loss for Vehicle Re-Identification

**DOI:** 10.3390/s20185142

**Published:** 2020-09-09

**Authors:** Xu Chen, Haigang Sui, Jian Fang, Mingting Zhou, Chen Wu

**Affiliations:** 1 The State Key Laboratory of Information Engineering in Surveying Mapping and Remote Sensing, Wuhan University, Wuhan 430079, China; 2010206190108@whu.edu.cn (X.C.); mintyzhou@whu.edu.cn (M.Z.); 2 College of Urban and Environmental Sciences, Central China Normal University, Wuhan 430079, China; fangjian06@whu.edu.cn; 3 National Engineering Research Center For E-Learning, Central China Normal University, Wuhan 430079, China; wuc_oct17@126.com

**Keywords:** sampling considering viewpoints, multi-proxy constraint loss, vehicle re-identification

## Abstract

Vehicle re-identification plays an important role in cross-camera tracking and vehicle search in surveillance videos. Large variance in the appearance of the same vehicle captured by different cameras and high similarity of different vehicles with the same model poses challenges for vehicle re-identification. Most existing methods use a center proxy to represent a vehicle identity; however, the intra-class variance leads to great difficulty in fitting images of the same identity to one center feature and the images with high similarity belonging to different identities cannot be separated effectively. In this paper, we propose a sampling strategy considering different viewpoints and a multi-proxy constraint loss function which represents a class with multiple proxies to perform different constraints on images of the same vehicle from different viewpoints. Our proposed sampling strategy contributes to better mine samples corresponding to different proxies in a mini-batch using the camera information. The multi-proxy constraint loss function pulls the image towards the furthest proxy of the same class and pushes the image from the nearest proxy of different class further away, resulting in a larger margin between decision boundaries. Extensive experiments on two large-scale vehicle datasets (VeRi and VehicleID) demonstrate that our learned global features using a single-branch network outperforms previous works with more complicated network and those that further re-rank with spatio-temporal information. In addition, our method is easy to plug into other classification methods to improve the performance.

## 1. Introduction

As people attach importance to traffic surveillance and public safety, there is an ever-increasing need to retrieve the same vehicles across cameras. Vehicle re-identification (Re-ID) aims to identify the same target vehicle in the large-scale gallery database, given a probe vehicle image. Some research solves this problem by license plate recognition [1,2]; however, it is difficult to get a clear shot of license plates in some views. Therefore, vision-based vehicle Re-ID has attracted more attention.

Compared with person Re-ID, vehicle Re-ID faces some unique challenges: (1) Different vehicle instances usually have highly similar appearance with those of the same type and color, regarded as inter-class similarity. (2) Images of the same vehicle captured from different cameras exhibit large variance in appearance due to different structures and details in different faces of the body, namely intra-class variance.

With regard to inter-class similarity, the methods of extracting partial discriminative region features [3,4] and generating similar anti-samples [5] have been put forward. Three even divisions to the global features were used to obtain local features in the Region-Aware deep model (RAM) [3] model. He et al. [4] detected special parts including lights, brands, windows, and jointed these local features with global features to improve the performance. Lou et al. [5] designed a distance adversarial scheme to generate similar hard negative samples, aiming at facilitating the discriminative capability. However, they neglected the influence of intra-class variance, resulting in the inability to learn a compact feature embedding space. In addition, detecting the pre-defined local regions requires additional training.

Some work [6,7,8,9,10,11] were devoted to addressing the intra-class variance problem of vehicle re-identification by predicting key points or viewpoints. The key points can be passed as input to feature extract network [9] or directly used as the discriminant regions to aggregate the orientation-invariant features [6] and trained supervised by IDs to distinguish similar vehicles [10]. Despite obtaining local discriminative features, key points require extra labels and are only partially visible in different viewpoints. The role of viewpoints can be divided into two categories: learning features [7,8] and learning metric [11]. For feature learning, inferring the multi-view feature [7] using the attention model and learning transformations of vehicle images between different viewpoints [8] are proposed. As for metric learning, Chu et al. [11] adopted different matrices to evaluate the similarity of vehicle images according to whether the viewpoints are similar. Again, these methods need additional labeling and prediction process. In addition to these methods, Bai et al. [12] performed an online grouping to cluster the similar viewpoints to optimize the distance metric. However, this network had a complicated training process.

In contrast to the above approaches, we propose a multi-proxy constraint loss (MPCL) function to deal with both intra-class variance and inter-class similarity problem in this paper. We introduce a novel sampling strategy considering viewpoints, which can help mine samples corresponding to different proxies in a mini-batch. A multi-proxy constraint loss function is implemented to learn multiple proxies of a class end to end without additional clustering and impose different constraints based on similarity, effectively achieving intra-class differentiation representation and a larger inter-class margin. We evaluate our approach on two large-scale vehicle Re-ID datasets, VeRi [13] and VehicleID [14] and compare the performance with other state-of-the-art vehicle Re-ID methods. Experimental results showed the superiority of our approach to multiple state-of-the-art vehicle Re-ID methods. The major contribution can be summarized as follows:

(1) We propose a novel sampling strategy considering different viewpoints, effectively selecting the samples captured by different cameras. This sampling strategy contributes to sample the images corresponding to different proxies in a mini-batch. Moreover, it helps to mine hard positive and negative sample pairs.
(2) A multi-proxy constraint loss function is implemented to learn the multiple intra-class proxies and constrain the distance to hard positive proxy less than to hard negative proxy. The feature embedding space supervised by this loss function is more compact, resulting in a larger inter-class distance.
(3) Our proposed approach can be seamlessly plugged into existing methods to improve performance with less effort. We conduct extensive experiments on two large-scale vehicle Re-ID datasets, achieving promising results.

The rest of this paper is organized as follows. Section 2 discusses related works of vehicle re-identification. Section 3 gives a detailed description of our proposed approach. Section 4 presents the implementation and evaluation of the experiments. Section 5 concludes this study.

## 2. Related Works

Research on vehicle Re-ID can be divided into two categories: one is view-independent, the other is based on multi-view. These view-independent methods concentrate on obtaining more robust features by aggregating multiple attributes or partial features. Cui et al. [15] fused the classification features of color, vehicle model, and pasted marks on windshield as the final features to describe the vehicle. Some studies used a variety of attributes to identify vehicles from coarse to fine, such as Progress Vehicle Re-identification (PROVID) [13] and RNN-based Hierarchical Attention (RNN-HA) [16]. These coarse-to-fine approaches require multiple recognition processes and cannot be implemented end to end. Because the differences between similar vehicles are mainly distributed in local regions, some work extracted partial features to improve discriminative ability. RAM [3] adopted horizontal segmentation to obtain local features. He et al. [4] introduced a detection branch to detect window, light, and brand, then combined these partial features and global features to help identify subtle discrepancies. However, these methods of using local features increase the complexity of the network and are usually difficult to train. In addition, the pre-defined areas cannot be detected on images captured from some views.

Taking into account the variance between different views of the same vehicle, some studies focus on generating multi-view features of the vehicle. Wang et al. [6] extracted features of 20 selected key points to aggregate the orientation-invariant feature. The Viewpoint-aware Attentive Multi-view Inference (VAMI) [8] model inferred multi-view features by adversarial training, after selecting core regions of different viewpoints by an attention model. Zhou et al. [7] used spatially concatenated multi-view images to train the network aiming at transforming a single-view image to multi-view features. Also, he proposed bi-directional Long Short-Term Memory (LSTM) units to learn successive transforms between adjacent views. Besides generating multi-view features, Chu et al. [11] learned two metrics for similar viewpoints and different viewpoints, then used the corresponding matric to evaluate the similarity of two images based on whether the viewpoints are similar. Tang et al. [9] reasoned the vehicle pose and shape with synthetic datasets and passed this information to the attributes and feature learning network. Khorramshahi et al. [10] increased a path to detect vehicle key points using the orientation as a conditional factor and extract the local features to distinguish similar vehicles. However, these multi-view approaches require additional labels of key points or viewpoints and complex training process.

In addition, the metric learning methods directly impose distance constraints on different classes and generally achieve good performance in face recognition [17,18,19,20,21,22,23] and person re-identification [24,25,26,27]. Therefore, some scholars also use metric learning to improve the performance of vehicle Re-ID. Liu et al. [14] used the cluster center instead of randomly selected anchor samples in order to solve the problem of the triplet loss which is sensitive to the selected anchor. Bai et al. [12] divided the same vehicle into different groups aiming at characterizing intra-class variance, and adopted an offline strategy to generate the center of the class and each group in this class. However, these center clustering methods require multiple computational processes. Chen et al. [28] designed the distance-based classification to maintain the consistency among criteria for similarity evaluation, but it does not solve the problem of intra-class variance.

## 3. The Proposed Method

The proposed method takes into account the impact of different viewpoints on the appearances of vehicles, and uses the viewpoint-based sampling strategy to better mine samples corresponding to different proxies. The feature embedding space is optimized by performing the multi-proxy constraint classification.

### 3.1. Sampling Strategy Considering Viewpoints

The appearances of the same vehicle captured by different cameras vary greatly. To address this problem, we design a novel sampling strategy considering multiple viewpoints. For vehicle Re-ID, the samples with large variance in the appearance usually have different viewpoints. Therefore, we select different vehicle images with different viewpoints for every identity to learn intra-class variance in each sampling.

**Figure 1:**
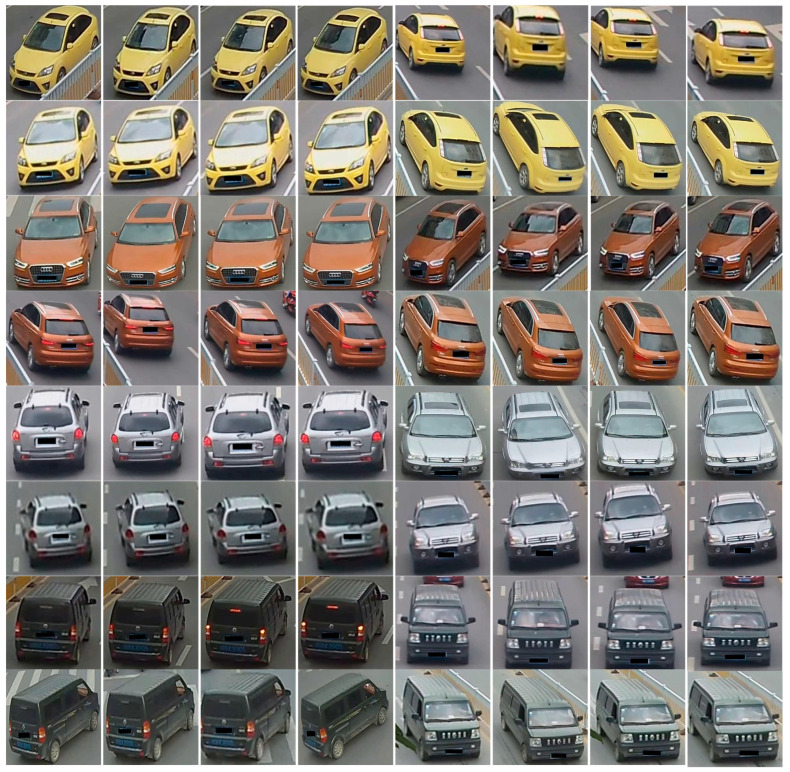
An example of samples using strategy considering viewpoints. *P*, *K*, and *V* are all set to 4. We can see that the images shot by the same camera are similar and images from different viewpoints show the intra-class variance.

In a mini-batch, we randomly sample P vehicle identities, and then randomly select K cameras for each identity. The vehicle images are randomly sampled V times under the restriction of both the identity and the camera. This strategy results in a mini-batch of P×K×V images, as shown in Figure 1.

Normally, an epoch is completed after all the vehicle identities are sampled. However, the distribution of vehicle images is not even, some identities have more images than others. If all identities are sampled in the same number, then many images are wasted in one epoch. Therefore, we perform N iterations in an epoch, and each iteration samples all the vehicle identities according to the above sampling strategy.

### 3.2. Multi-Proxy Constraint Loss

Considering the intra-class variance and inter-class similarity, we design a multi-proxy constraint loss function to learn the multiple proxies for one class end to end. The proxy is the center vector of the class. Unlike the usual practice of using a center vector to represent a class, we use multiple center vectors to represent a class. Instead of using extra clustering [12], we adopt a full connected (FC) layer to learn the multiple proxies for each class. The weight vectors of this FC layer are regarded as the proxies of all the classes, and the size is determined by the number of classification and proxies in each class. The weight matrix W is expressed as $\left[w_{11},w_{12},\ldots w_{1m},w_{21},w_{22},\ldots w_{2m},\ldots w_{c1},w_{c2},\ldots w_{cm}\right]$, while there are *c* classes in total and each class has *m* proxies. We compute the cosine similarity between the feature f and the weight matrix W as follows,

(1) $$S_{i,j,k}=f{\left(x_{i}\right)}^{T}w_{j,k}/(||f(x_{i})||*||w_{j,k}||),$$

In this way, the size of cosine similarity is c×m, but the labels for supervision are c. To constrain every *m* weights adjacent to represent multiple proxies of the same class, the minimum value in $\left[s_{i,j,1},s_{i,j,2},\ldots s_{i,j,m}\right]$ is used as the similarity between feature $f(x_{i})$ and class i, and the maximum value in $\left[s_{i,j,1},s_{i,j,2},\ldots s_{i,j,m}\right]$ is taken as the similarity between feature $f(x_{i})$ and class j, while i is not equal to j.

(2) $$S_{i,i}=\underset{k=1\ldots m}{\min}S_{i,i,k},$$

(3) $$S_{i,j,i\neq j}=\underset{k=1\ldots m}{\max}S_{i,j,k},$$

Then we get the prediction probability after normalizing the cosine similarity by SoftMax function. The loss function for a mini-batch is computed as:

(4) $$L_{mcs}=\sum_{i}-\log(\frac{e^{S_{i,j}}}{\sum_{j}e^{S_{i,j}}}),$$

**Figure 2:**
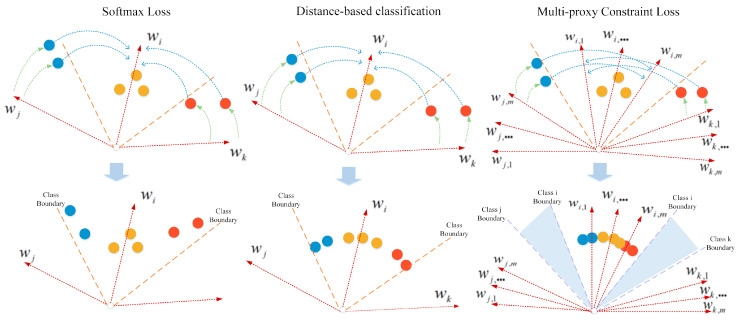
The comparison of optimization process supervised by SoftMax loss, distance-based classification, and multi-proxy constraint loss. The blue arc means the samples are pull closer to the weight vector of the corresponding class, and the green arc shows the samples are push away from the weight vector of the neighboring class. It can be seen that the multi-proxy constraint loss will generate a margin between adjacent classes.

Compared with SoftMax loss and distance-based classification [28], the multi-proxy constraint loss has a different optimization process, as illustrated in Figure 2. SoftMax loss aims to pull all the positive samples within the boundaries of the class. Distance-based classification eliminates the effect of the length of feature vector and makes the classification consistent with the final similarity evaluation criteria. Multi-proxy constraint loss goes a step further based on distance-based classification, requiring that the distance to the furthest intra-class proxy is less than the distance to the closest inter-class proxy. With the supervision of the multi-proxy constraint loss, the embedding space is more compact within classes, as well as having a larger inter-class distance. 

### 3.3. Network Architecture

**Figure 3:**
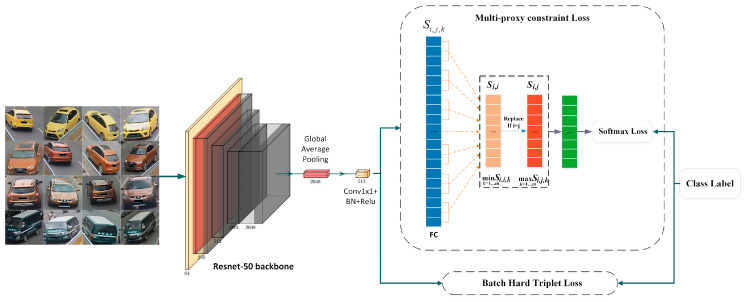
The proposed network architecture. We add a block consisting of Conv1x1, BatchNorm, and Rectified Linear Unit (ReLU) to reduce the dimension of the features from 2048 to 512. The multi-proxy constraint loss function and the batch hard triplet loss function are jointed to learn the feature embedding space.

As show in Figure 3, we adopt a pre-trained network with partial modification for the vehicle Re-ID task. ResNet-50 [29] is adopted as the backbone, as it achieved competitive performance in some Re-ID works. The structure before the original global average pooling (GAP) layer shares the similar architecture with the backbone, except for that the down-sample stride is changed to 1 in res_conv5_1 block in order to increase the output resolution.

A reduction-dim block is added to force the network to learn the discriminative features with fewer channels. The reduction-dim block consists of three layers. A 1×1 convolution layer with a batch normalization and Rectified Linear Unit (ReLU) reduces the 2048-dim feature to the 512-dim feature.

The multi-proxy constraint loss and batch hard triplet loss [24] together constitute the final loss function. Based on our proposed sampling strategy, the batch hard triplet loss function for a mini-batch is defined as follows:

(5) $$L_{bht}=\max\{\sum_{i=1}^{P}\left[mg+\underset{\begin{array}{l} j=1\ldots P\\ n_{1}=1\ldots K*V\\ n_{2}=1\ldots K*V\\ j\neq i \end{array}}{\max}D(f(x_{n_{1}}^{i}),f(x_{n_{2}}^{j}))-\underset{\begin{array}{l} n_{1}=1\ldots K*V\\ n_{2}=1\ldots K*V \end{array}}{\min}D(f(x_{n_{1}}^{i}),f(x_{n_{2}}^{i}))\right],0\},$$

where mg is the margin, f is the feature learned by the network, $x_{n_{1}}^{i}$ and $x_{n_{2}}^{i}$ correspond to the *n*_1_-th and *n*_2_-th images for the *i*-th vehicle identity, and *D* stands for the cosine function. All the input features are normalized.

The overall loss function is formulated as follows:

(6) $$L=\lambda_{1}L_{mcs}+\lambda_{2}L_{bht},$$

where $\lambda_{1}$, $\lambda_{2}$ denote the weights of the corresponding loss. For simplicity, we set all the weights to one. With the strong constraint on distance between different vehicle identities by batch hard triplet loss, the multi-proxy constraint loss is fast in convergence. During testing phases, the 512-dim feature before the classification layer is used as the final descriptor for the image.

## 4. Experiments

In this section, we evaluate the performance of our proposed approach on two large-scale vehicle Re-ID datasets. The effectiveness of the multi-proxy constraint loss function and the influence of the parameter and sample strategy are investigated.

### 4.1. Implementation Details

Our network is implemented on the PyTorch framework. The backbone network, Resnet-50, is pre-trained on ImageNet. The input images are resized to 256×256. When training, we perform random horizontal flipping and random erasing on the training dataset for data augmentation. The sampling parameters *P*, *K*, and *V* are all set to 4, so the mini-batch size is 64. The iteration number N in an epoch is set to 10 for VeRi-776 [30] dataset as each vehicle has multiple images captured by one camera in this dataset, while N is set to 1 for VehicleID [14] dataset since the vast majority of vehicle identities in this dataset have fewer than 16 images. The proxy number m is set to 8 and 2, respectively, for VeRi-776 and VehicleID datasets, because images in VeRi-776 dataset were taken at different viewpoints and images in VehicleID have only the front and back views. In batch hard triplet loss function, the margin m is set to 0.3. We adopt the SGD optimizer with the momentum of 0.9. A warming-up strategy [27] is used to help the network initialize better before applying a large learning rate. The initial learning rate is $1\times 10^{-4}$, and the learning rate increases linearly to $10^{-3}$ within 10 epochs. The learning rate at epoch 10 is set to $10^{-3}$, then decreased to $10^{-4}$ at epoch 60 respectively. The total epoch number of all the experiments is 100. When evaluating, we average the features of the original image and the horizontal flipped one as the final feature. This is the usual practice of obtaining more robust features in person Re-ID.

### 4.2. Datasets and Evaluation Metrics

We evaluate our proposed approach on two large-scale vehicle Re-ID datasets, VeRi-776 [30] and VehicleID [14]. The details of these two datasets are as follows:

VeRi-776 is a dataset containing multi-view vehicle images. It has a total of 776 vehicle identities from 20 cameras in real-world traffic surveillance environment. In addition to vehicle ID and camera ID, the colors, types, and spatio-temporal information are provided. 576 vehicles are used for training, and the remaining 200 vehicles for testing. 1678 images from the test vehicle images are selected as query images. Compared to VeRi, the images in VehicleID dataset have only the front and rear viewpoints. 110,178 images of 13,134 vehicles are used for training and 111,585 images of 13,133 vehicles for testing. Three subsets are proposed as test dataset of different scales, with 800, 1600, and 2400 vehicles, respectively.

The mean average precision (mAP) and cumulative match characteristics (CMC) are adopted to evaluate the performance, which are the same evaluation criteria with previous work. The CMC curve shows the probability that the query identity appears in different-sized search lists. The CMC at Top-k can be defined as:

(7) $$CMC@k=\frac{\sum_{q=1}^{Q}gt(q,k)}{Q},$$

where Q is the number of queries and gt(q,k) equals one if q appears in the Top-k of the ordered list. The *CMC* evaluation requires that the number of the ground-truth image for a given query should be one.

The mAP metric evaluates the accurate of the overall predications. *AP*(*q*) for the query image *q* is calculated as:

(8) $$AP(q)=\frac{\sum_{k=1}^{n}P(k)\times gt(k)}{N_{gt}(q)},$$

where *n* and $N_{gt}(q)$ are the numbers of retrieved vehicles and true retrievals for q respectively. P(k) is the precision at cut-off of k images, gt(k) indicates whether each recall image is correct or not. The mAP is calculated as:

(9) $$mAP=\frac{\sum_{q}AP(q)}{Q},$$

where Q is the total number of queries.

### 4.3. Comparisons to the State-of-the-Art

We compare our proposed approach with state-of-the-art vehicle Re-ID methods on the two above-mentioned datasets.

#### 4.3.1. Performance Comparisons on VeRi-776 Dataset

Recent works on vehicle Re-ID can be divided as viewpoint-independent and viewpoint-dependent. The viewpoint-independent methods such as PROVID [13], RHH-HA [16], RAM [3], Multi-Region Model (MRM) [31] mainly focus on learning of robust global and local features or learning of distance metric (Batch Sample (BS) [32] and Feature Distance Adversarial Network (FDA-Net) [5]). The viewpoint-dependent methods are dedicated to learning orientation-invariance features (Orientation Invariant Feature Embedding (OIFE) and OIFE + ST) [6] or multi-view features (VAMI and VAMI + ST) [8]. We group the comparison methods based on whether or not the viewpoint information is used.

Table 1 shows the results on VeRi dataset. This dataset provides the camera IDs and shooting time, so we use the camera ID to distinguish viewpoints when sampling. In addition, there are some methods using the spatio-temporal information to improve the performance of vehicle Re-ID, for example, OIFE [6], VAMI [8] and Pose Guide Spatiotemporal model (PGST) [33]. The annotations used by these methods are also outlined. Our proposed method outperforms all these methods even if those approaches use extra attributes or none-visual cues. Compared with other methods using viewpoints, such as VAMI [8], we use camera ID to simply distinguish views without additional annotation, but our MPCL exceeds the former method by 17.33% mAP. Although the Pose-Aware Multi-Task Re-Identification (PAMTRI) [9] adopts a more complicated backbone DenseNet201 and trains with both real and synthetic data, our approach outperforms PAMTRI by 6.77% mAP, 3.45% Rank-1 and 1.36% Rank-5.

#### 4.3.2. Performance Comparisons on VehicleID Dataset

According to the evaluation protocol proposed by Liu et al. [14], we provide results on three different test datasets (i.e., small, medium and large with testing size = 800, 1600, 2400), as shown in Table 2. Without camera IDs, we randomly select 16 images for each identity, the rest remains unchanged. From the results, the use of multi-proxy constraint loss function can achieve the best performance at Rank-1 and Rank-5 on the three test datasets compared to other state-of-the-art methods. Even without viewpoints, our proposed loss function contributes to better feature embedding by learning multiple intra-class proxies.

### 4.4. Ablation Analysis

We conduct ablation study on VeRi dataset to verify the effectiveness of the sampling strategy and view-aware distance loss function and examine the influence of some parameters.

#### 4.4.1. The Validation of Multi-Proxy Constraint Loss

The multi-proxy constraint loss is a polycentric classification based on the cosine distance and SoftMax, so we compare the retrieval performance under the supervision of SoftMax and distance-based classification. As shown in Table 3, multi-proxy constraint loss function outperforms SoftMax loss function and distance-based classification. All the three methods are trained under the joint supervision of batch hard triplet loss function, adopting the same network and learning parameters. Multi-proxy constraint loss beats distance-based classification by 1.34% for mAP on VeRi-776, and surpasses distance-based classification and SoftMax loss in the three different scale datasets of VehicleID. This shows that the multi-proxy constraint loss function effectively pulls closer the features from the same class and pushes away those from different classes.

**Figure 4:**
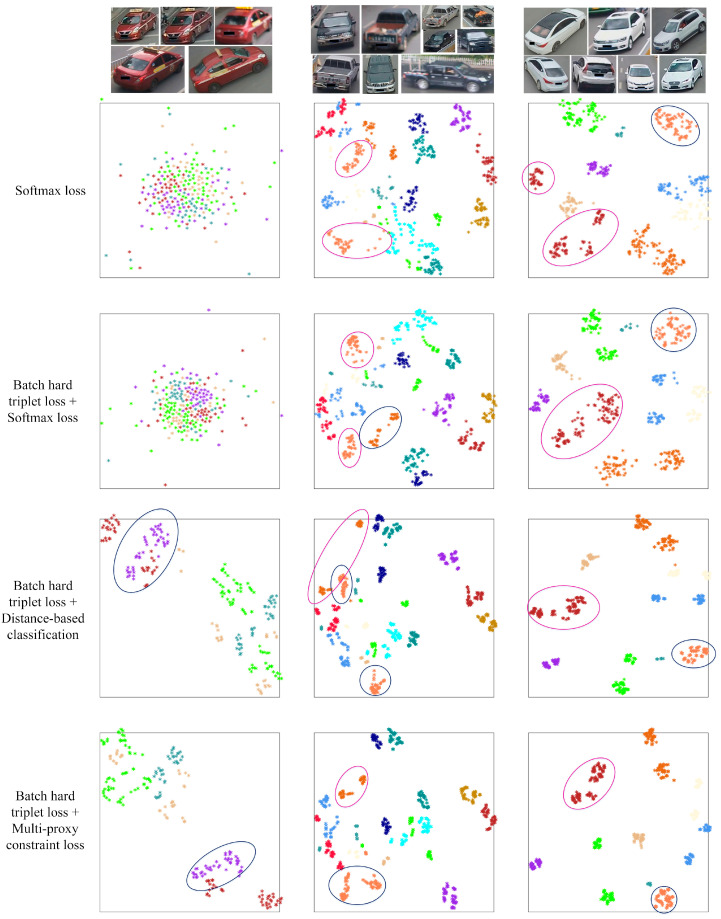
Visualization of the feature distribution by t-SNE on VeRi-776 test dataset. Different colors represent different vehicles. We randomly select three sets of similar images for comparison. The last two row shows the results of distance-based classification and multi-proxy constraint loss from top downwards, respectively. Compared with distance-based classification, the multi-proxy constraint loss contributes to a more compact feature embedding space.

The feature distribution by t-SNE on VeRi-776 test dataset is show in Figure 4. We compare three sets of the feature embedding space, all of which consist of similar samples. Compared to the distribution supervised by distance-based classification, there are larger inter-class margins and smaller intra-class distances in feature embedding space learned by multi-proxy constraint loss. It can be clearly seen that under the supervision of multi-proxy constraint loss, the distribution within the class colored with purple in the first group is more compact, especially it has a greater inter-class distance to the class colored with dark red. In the second group, the intra-class distance of the class colored with light red is greatly reduced under the constraint of multi-proxy loss, while the class colored with light red learned by distance-based classification is separated by other classes. All the classes learned from multi-proxy constraint can be better separated from each other.

**Figure 5:**
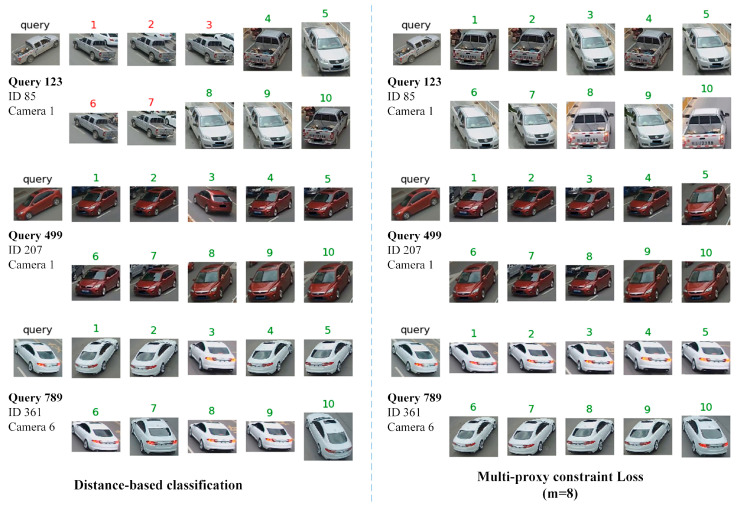
The Top-10 retrieval results on VeRi-776 dataset. The left-most images labeled ‘query’ are the query images. The two columns are the results of distance-based classification and multi-proxy constraint loss (m = 8). The ground-truth is labeled with green number. The wrong images will be labeled with red numbers.

Figure 5 visualizes the Top-10 retrieval results of distance-based classification and multi-proxy constraint loss for three query images. For query 123, multi-proxy constraint loss lists the positive top 10 images, but the negative similar images in the list obtained by distance-based classification are ranked higher. From the rank lists, we can see the features of images with similar viewpoints cluster together supervised by multi-proxy constraint loss, while the features learned by distance-based classification do not show this pattern. Although the top 10 images queried for id 499 and 789 by both methods are all the positive ones, the ranking results of multi-proxy constraint loss are more in line with the criteria for manual discrimination, i.e., the similarity of images with similar viewpoints is higher. This also shows that intra-class clustering can be effectively achieved by multi-proxy constraint loss, making the learned feature representation better deal with the problem of large intra-class variance.

#### 4.4.2. The Influence of the Number of Proxies

The only hyper-parameter the multi-proxy constraint loss function brings is *m*, which indicates the number of proxies. For the multi-proxy constraint loss, a key step is to determine the number of centers. Too few centers may lead to poor intra-clustering results. However, the larger *m*, the fully connected layer representing multiple proxies of all the classes has more parameters, making the network more difficult to converge. To verify the effect of the number of proxies, we train the proposed method with different *m* and compare the evaluation results on VeRi-776 dataset.

**Figure 6:**
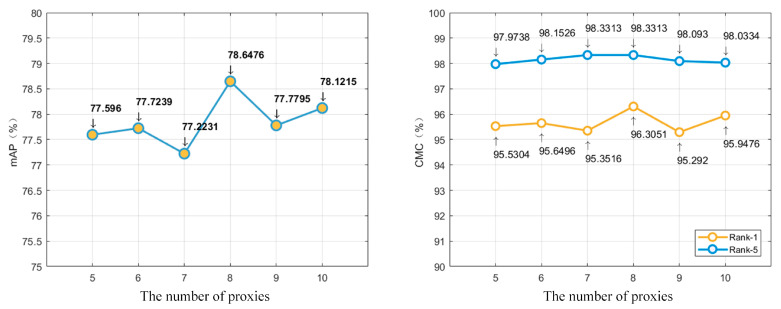
The comparison of different number of proxies on VeRi-776 dataset. The chart on the left shows the score of mAP, and the chart on the right corresponds to the scores of Rank-1 and Rank-5.

As shown in Figure 6, the best scores of Rank-1, Rank-5, and mAP were obtained when setting *m* to 8. We infer that intra-clustering can be better performed with 8 proxies, because the shooting angle for the same vehicle can be roughly divided into 8 directions including front, left front, left, left rear, rear, right rear, right, right front. In addition, the network with an even number of proxies performs better than that with its adjacent odd number of proxies.

#### 4.4.3. The Influence of Sampling Strategy

To verify whether our proposed sampling strategy contributes to learn multiple intra-class proxies, we compare the performance of two sampling strategies, as shown in Table 4. Both two sampling strategies have 4 vehicle identities in a mini-batch, and each identity has 16 images. The only difference between these two sampling strategies is whether the camera information is taken into consideration when sampling images for each vehicle. The sampling strategy considering viewpoints achieves better performance. This indicates that selecting samples from different viewpoints can effectively enrich the diversity of samples in a mini-batch and promote the network to better learn multiple intra-class proxies. Moreover, sampling considering viewpoints helps to mine hard positive and negative sample pair in a mini-batch.

## 5. Conclusions

In this paper, we propose a sampling strategy considering viewpoints and the multi-proxy constraint loss function that deals with the intra-class variance and inter-class similarity problems for vehicle Re-ID. This sampling strategy is beneficial for better learning multiple intra-class proxies. With this sampling strategy, the multi-proxy constraint loss function effectively uses the most difficult positive and negative proxies to impose stronger constraint on samples, leading to large inter-class margins and small intra-class distances. Experiments on two large-scale vehicle datasets demonstrate the superiority of our method. In particular, our approach achieves state-of-the-art performance on VeRi-776 dataset, with 78.65% mAP, 96.31% Rank1 and 98.33% Rank5. In addition, our proposed multi-proxy constraint loss function also works for other classification tasks and easy to plug into other frameworks to improve the performance.

However, the relationship between samples from different viewpoints with the same identity is neglected, which helps to identify accurately when faced with large variance in appearance. In the future, we will consider facilitating graph convolutional neural networks to learn the relationship between samples to obtain more robust features.

## Figures and Tables

**Table 1:** Comparison of mAP (%) and match rate (CMC) at Top-k (%) on VeRi-776 dataset.

Model	Annotations	Rank-1	Rank-5	mAP
PROVID [13] ^1^	ID + Attribute + Plate	61.44	78.78	27.77
RHH-HA [16]	ID + Attribute	74.79	87.31	56.80
Path-LSTM [34] ^1^	ID	83.49	90.04	58.27
RAM [3]	ID + Attribute	88.60	94.00	61.50
BS [32]	ID	90.23	96.42	67.55
FDA-Net [5]	ID + Attribute	84.27	92.43	55.49
MRM [31]	ID	91.77	95.82	68.55
AFL [35]	ID + Attribute	86.29	94.39	57.43
PGST [33] ^1^	ID + Pose + CameraID	72.42	91.25	54.15
PGST + visual-SNN [33] ^1^	ID + Pose + CameraID	89.36	94.40	69.74
OIFE [6]	ID + Keypoints	89.43	-	48.00
OIFE + ST [6] ^1^	ID + Keypoints	92.35	-	51.42
VAMI [8]	ID + Attribute	77.03	90.82	50.13
VAMI + ST [8] ^1^	ID + Attribute	85.92	91.84	61.32
PAMTRI [9]	ID, Key points, Attribute	92.86	96.97	71.88
AAVER (ResNet-50) [10]	ID, Key points	88.97	94.70	58.52
MPCL (Ours)	ID + CameraID	**96.31**	**98.33**	**78.65**

Rank-K represents the probability that at least one true positive is ranked within the Top-k positions of search lists. 1 indicates that the spatio-temporal information is used in this approach.

**Table 2:** Results of Top-k metric (%) on VehicleID dataset.

Model	Annotations	Small		Medium		Large	
		Rank1	Rank5	Rank1	Rank5	Rank1	Rank5
CCL [14]	ID + Attribute	49.00	73.50	42.80	66.80	38.20	61.60
C2F [36]	ID + Attribute	61.10	81.70	56.20	76.20	51.40	72.20
RAM [3]	ID + Attribute	75.20	91.50	72.30	87.00	67.70	84.50
GSTE [15]	ID	75.90	84.20	74.80	83.60	74.00	82.70
FDA-Net [5]	ID + Attribute	64.03	82.8	57.82	78.34	49.43	70.48
OIFE [6]	ID + Keypoints	-	-	-	-	67.00	82.90
VAMI [8]	ID + Attribute	63.12	83.25	52.87	75.12	47.34	70.29
MPCL (Ours)	ID	**81.75**	**92.63**	**78.79**	**90.71**	**75.91**	**88.91**

CCL represents coupled cluster loss, C2F represents coarse to fine rank loss, GSTE represents group sensitive triplet embedding.

**Table 3:** Performance comparison on the VeRi-776 and VehicleID datasets.

Dataset		Method	Rank-1	Rank-5	Rank-10	mAP
VeRi-776		SoftMax—	94.46	97.68	98.45	76.58
		Distance-based classification	95.83	98.03	98.57	77.31
		Multi-proxy constraint	**96.31**	**98.33**	**98.75**	**78.65**
VehicleID	Small	SoftMax—	79.63	90.69	94.25	68.01
		Distance-based classification	80.88	91.13	93.69	71.30
		Multi-proxy constraint	**81.75**	**92.63**	**95.75**	**72.31**
	Medium	SoftMax—	74.96	88.50	92.75	62.28
		Distance-based classification	78.50	89.67	93.75	67.02
		Multi-proxy constraint	**78.80**	**90.71**	**94.21**	**67.98**
	Large	SoftMax—	71.63	86.47	90.53	57.80
		Distance-based classification	75.00	88.47	92.06	62.45
		Multi-proxy constraint	**75.91**	**88.91**	**93.10**	**63.70**

**Table 4:** Performance from different sampling strategies on the VeRi-776 dataset. The best performance is bold.

Method	Rank-1	Rank-5	mAP
Sampling considering viewpoints	**96.31**	**98.33**	**78.65**
Random sampling	95.41	97.56	77.72
